# Endoscopically Assisted Periacetabular Osteotomy for Treatment of Hip Dysplasia: The Orthopaedic Total Extraperitoneal Approach

**DOI:** 10.1002/atn2.70427

**Published:** 2026-11-09

**Authors:** Vedat Öztürk, Malik Çelik, Salih Çağdaşer, Önder Murat Hürmeydan, Furkan Çağlayan Aslantaş, Cemal Kural, Mustafa Gökhan Bilgili

**Affiliations:** ^1^ Department of Orthopedics and Traumatology Bakırköy Dr. Sadi Konuk Training and Research Hospital Istanbul Turkey

## Abstract

The orthopaedic total extraperitoneal (O‐TEP) approach is an endoscopic technique that has recently emerged as an option, particularly for the surgical management of pelvic and acetabular fractures. Unlike conventional orthopaedic endoscopic procedures, O‐TEP was developed by adapting the laparoscopic totally extraperitoneal approach, commonly used for inguinal hernia repair, to orthopaedic surgery. In technically demanding procedures such as periacetabular osteotomy (PAO), minimizing soft‐tissue dissection and directly visualizing critical neurovascular structures are essential. Owing to its minimally invasive design, the O‐TEP PAO technique is intended to limit soft‐tissue dissection while enabling direct visualization of key vascular and neural structures at risk. This Technical Note provides a step‐by‐step description of O‐TEP PAO technique for acetabular dysplasia and discusses its feasibility, potential advantages, and key limitations in the context of the current literature on endoscopy‐assisted PAO techniques.

## Graphical abstract

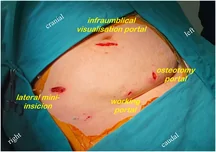

**VIDEO 1:** The O‐TEP PAO technique for the treatment of symptomatic right acetabular dysplasia in a 36‐year‐old female patient positioned supine. Endoscopic visualization is obtained through the infraumbilical visualization portal. The video shows preparation of the preperitoneal workspace through the O‐TEP approach, endoscopic identification and protection of the obturator neurovascular bundle and external iliac vessels, and stepwise completion of the ischial and pubic osteotomies under combined endoscopic and fluoroscopic guidance. The posterior column and supra‐acetabular iliac osteotomies are subsequently performed through a lateral mini‐incision, followed by acetabular fragment reorientation and definitive screw fixation. O‐TEP, orthopaedic total extraperitoneal; PAO, periacetabular osteotomy. Video content can be viewed at https://doi.org/10.1002/atn2.70427.

Periacetabular osteotomy (PAO) is technically demanding because it requires multiple bone cuts around the acetabulum in close proximity to critical neurovascular structures.^1^, ^2^ Several surgical modifications—including endoscopy‐assisted PAO, combined hip arthroscopy and PAO, and open intrapelvic approaches—have been described to reduce surgical morbidity and improve visualization of neurovascular structures at risk.^3^, ^4^, ^5^, ^6^, ^7^, ^8^, ^9^ However, previously described endoscopy‐assisted PAO techniques have primarily focused on visualizing some of these structures and reducing the size of conventional surgical incisions; the osteotomies themselves were performed through these smaller open incisions rather than through endoscopic portals.^6^, ^10^ Open intrapelvic approaches provide broader and more direct visualization of the anterior intrapelvic anatomy but still require open surgical exposure.^5^, ^11^

The orthopaedic total extraperitoneal (O‐TEP) approach adapts the laparoscopic totally extraperitoneal approach to orthopaedic surgery, providing direct access to the anterior intrapelvic space and quadrilateral surface with limited soft‐tissue dissection.^12^, ^13^, ^14^ In this article, we describe the O‐TEP PAO technique, in which the ischial and pubic cuts of a standard Bernese PAO are performed through a portal‐based extraperitoneal workspace under direct endoscopic visualization, allowing identification and protection of critical neurovascular structures. The posterior column and supra‐acetabular iliac cuts are subsequently completed through a limited lateral mini‐incision, preserving the standard Bernese PAO configuration while minimizing open surgical exposure.

## SURGICAL TECHNIQUE

The procedure was performed by a senior orthopaedic surgeon with at least 4 years of experience in endoscopic orthopaedic pelvic surgery and at least 3 years of experience in PAO. Before initiating endoscopic pelvic surgery, the surgeon received hands‐on training in laparoscopic totally extraperitoneal approach by attending procedures performed by general surgeons. The procedure was performed using the previously described O‐TEP approach, adapted from the laparoscopic totally extraperitoneal approach for orthopaedic surgery.^12^, ^13^, ^14^ The surgical technique is illustrated step by step using intraoperative endoscopic images and video recordings, intraoperative surgical video footage, and fluoroscopic images (Figures 1, 2, 3, 4, 5, 6, 7, 8, Video 1).

**FIGURE 1:**
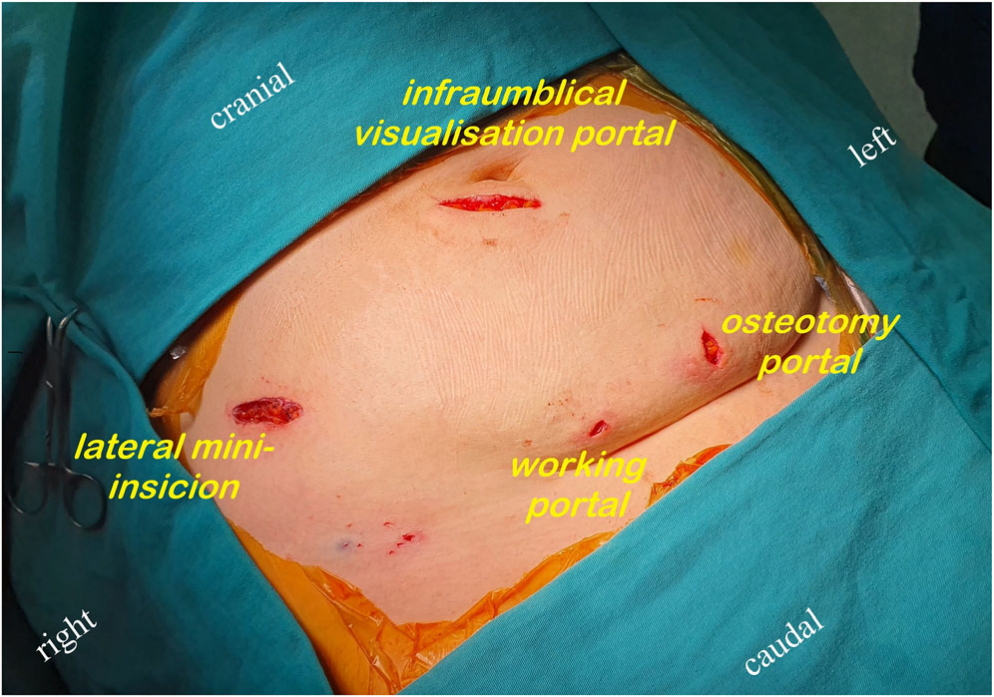
Skin incisions used in the O‐TEP PAO technique. The patient is positioned supine, with the right hip as the operative side. The infraumbilical visualization portal, working portal, osteotomy portal, and lateral mini‐incision are shown. (O‐TEP, orthopaedic total extraperitoneal; PAO, periacetabular osteotomy.)

**FIGURE 2:**
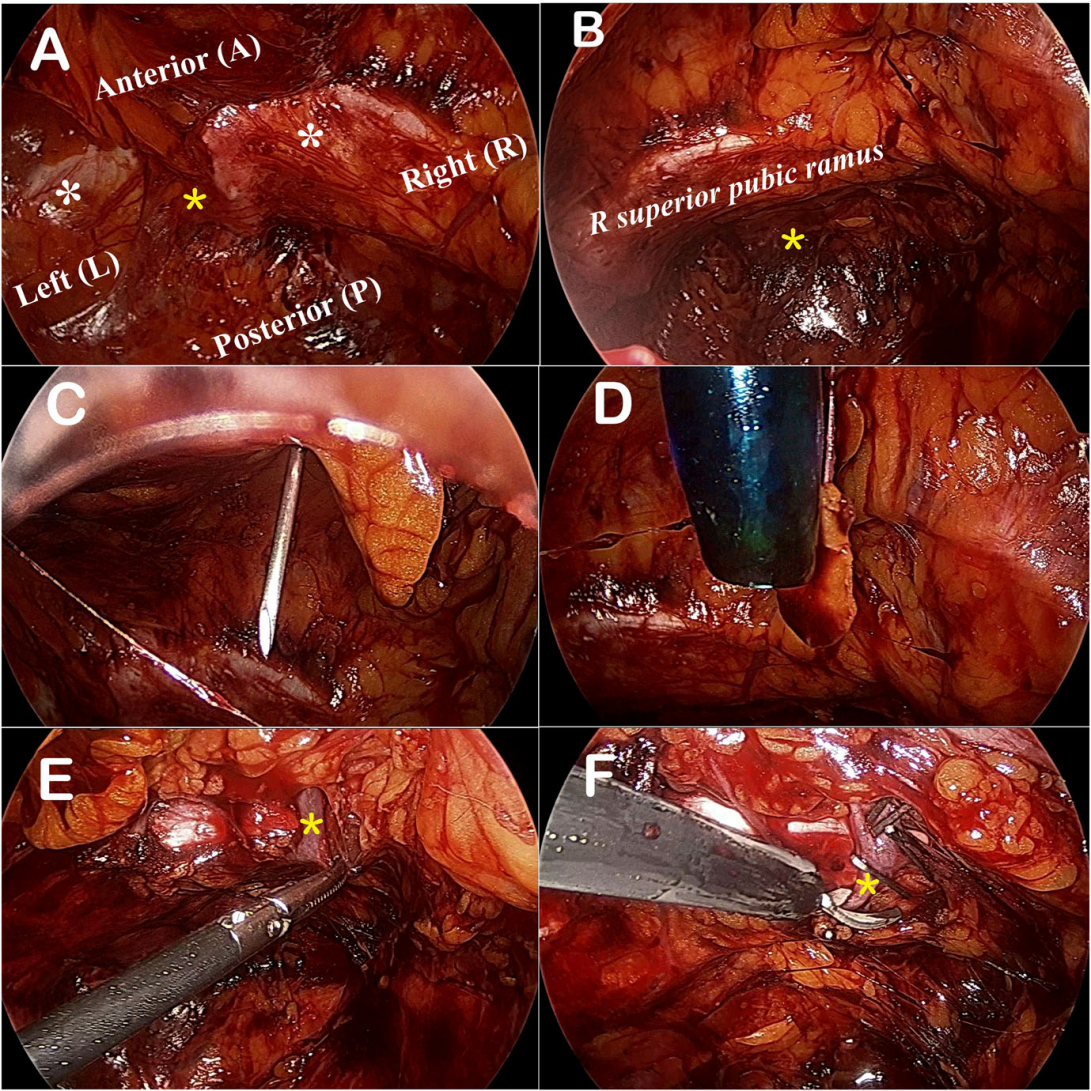
Endoscopic views obtained through the infraumbilical visualization portal with the patient in the supine position and the right hip as the operative side. (A) Anterior pelvic ring showing the pubic symphysis (yellow *) and bilateral superior pubic rami (white *). (B) Right superior pubic ramus and underlying obturator foramen (yellow *). (C) Percutaneous localization needle confirming the portal entry point and trajectory. (D) Placement of the 5‐mm working trocar. (E) Dissection of the corona mortis (yellow *). (F) Ligation and division of the corona mortis with endoscopic scissors (yellow *).

**FIGURE 3:**
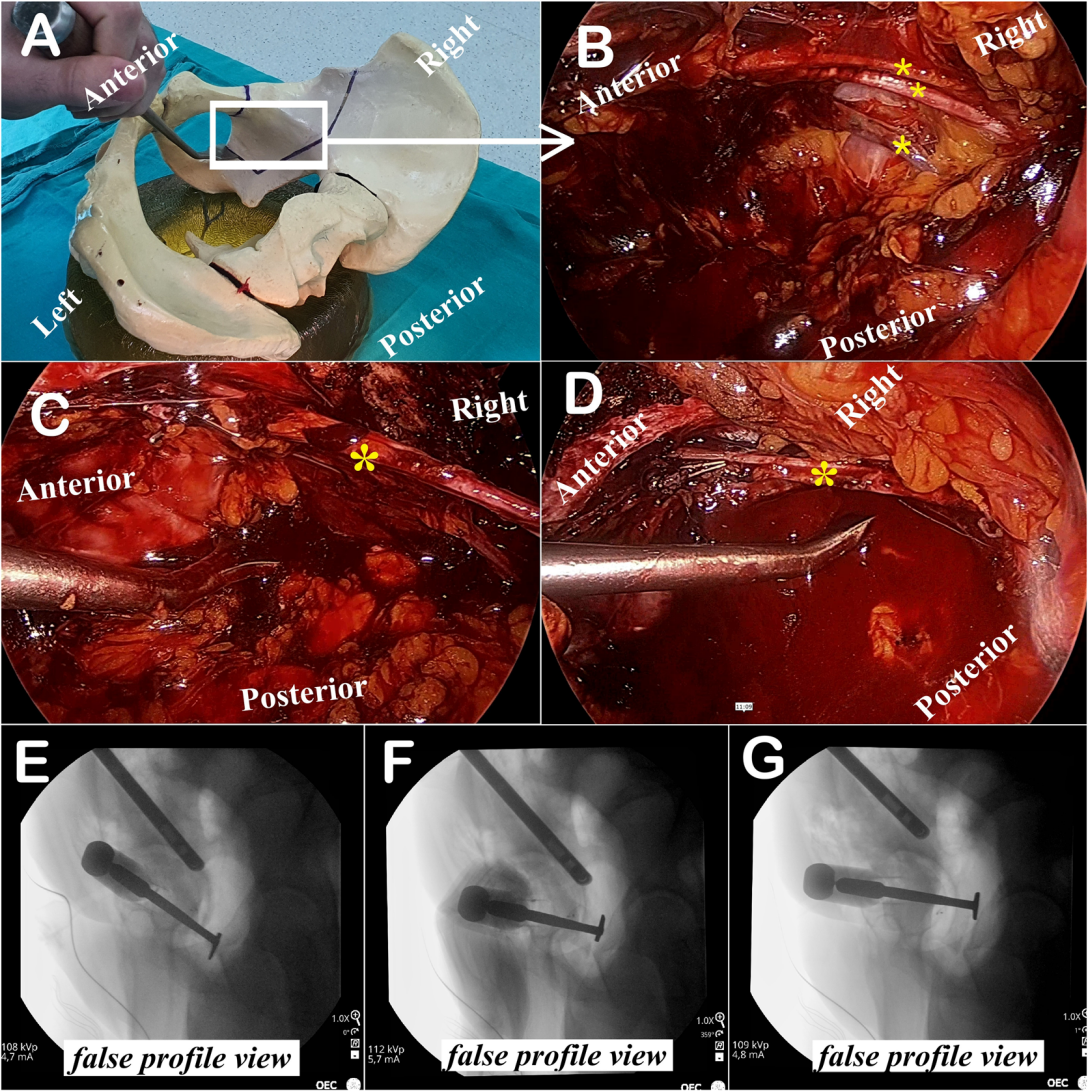
Ischial osteotomy. Endoscopic views obtained through the infraumbilical visualization portal with the patient in the supine position and the right hip as the operative side. (A) Presentation of the ischial osteotomy on a synthetic pelvic model. (B) Endoscopic view of the obturator neurovascular bundle, showing the obturator nerve, artery, and vein from superior to inferior (yellow *). (C,D) Transmuscular placement of the osteotome through the m. obturator internus, inferior to the obturator neurovascular bundle (yellow *), under direct endoscopic visualization and stepwise advancement of the cut posteriorly. (E‐G) Sequential fluoroscopic views showing progression of the ischial osteotomy.

**FIGURE 4:**
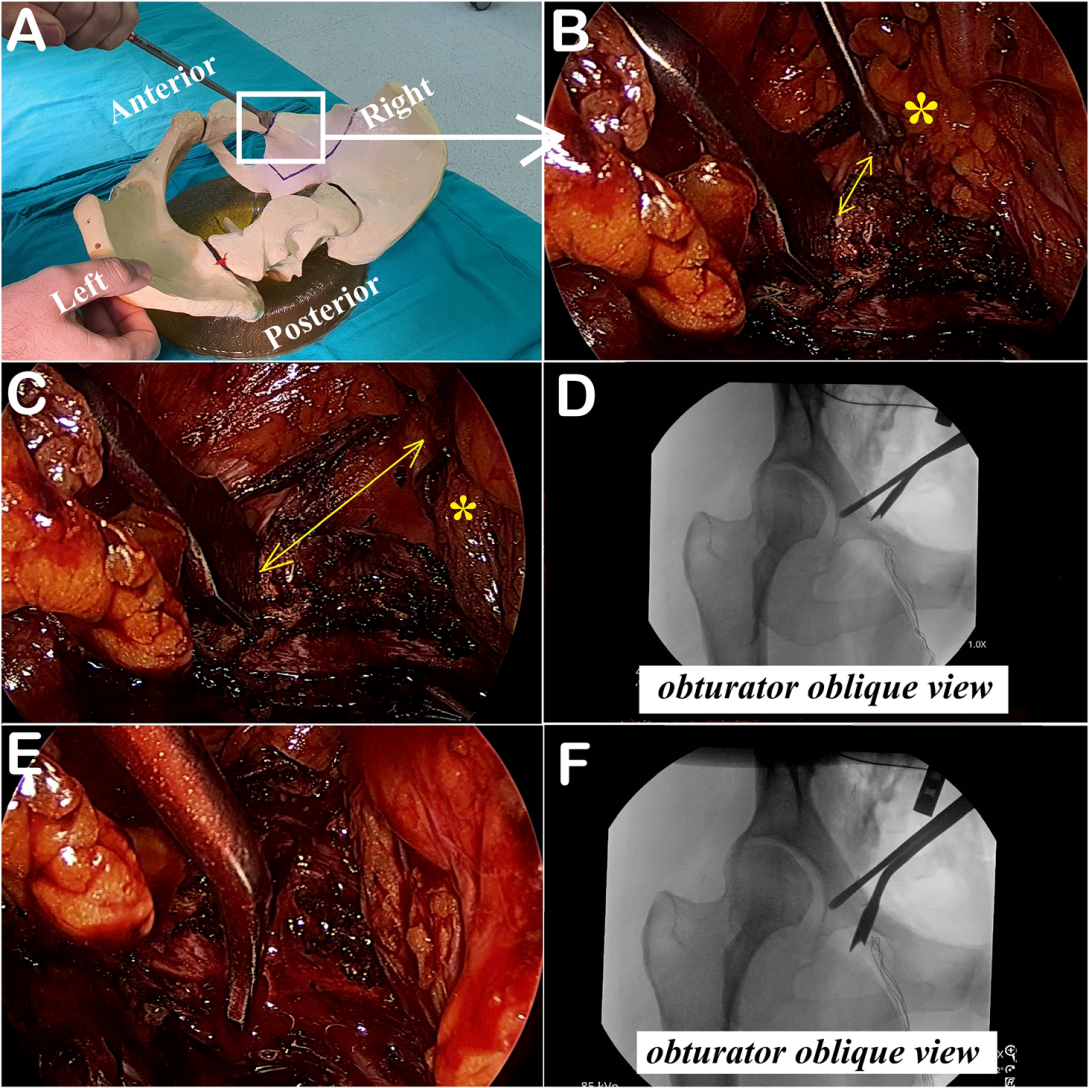
Pubic osteotomy. Endoscopic views obtained through the infraumbilical visualization portal with the patient in the supine position and the right hip as the operative side. (A) Presentation of the pubic osteotomy on a synthetic pelvic model. (B,C) Lateral retraction of the external iliac vessels (yellow *) with a blunt retractor and safe positioning of the osteotome under direct endoscopic visualization. (D) Fluoroscopic confirmation of the osteotome position. (E) Stepwise advancement of the pubic osteotomy. (F) Fluoroscopic view showing progression of the pubic osteotomy.

**FIGURE 5:**
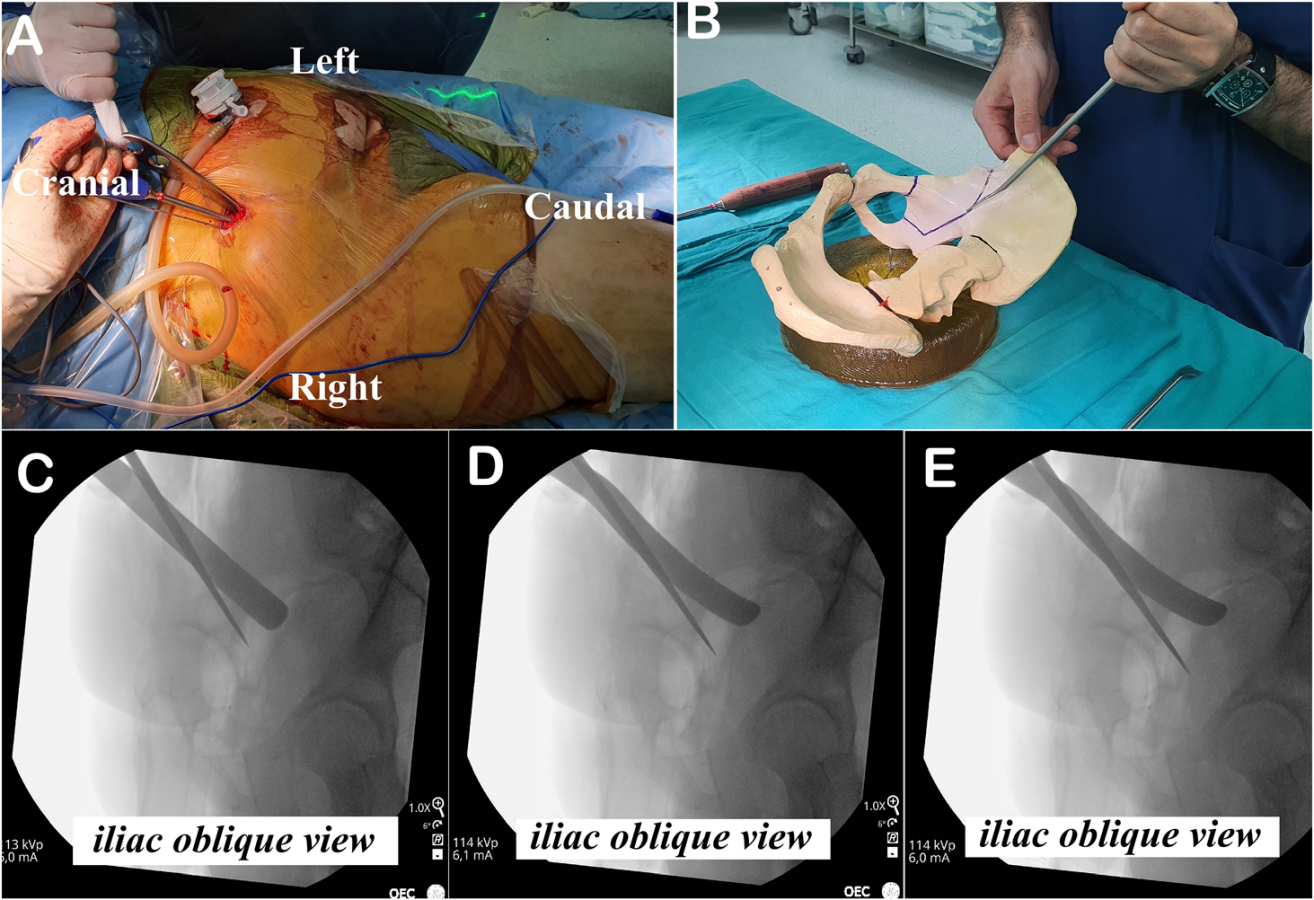
Posterior column cut with the patient positioned supine and the right hip as the operative side. (A) Soft‐tissue retraction with Hohmann retractors and positioning of the osteotome. (B) Presentation of the posterior column cut on a synthetic pelvic model. (C‐E) Sequential fluoroscopic views showing stepwise progression of the posterior column cut.

**FIGURE 6:**
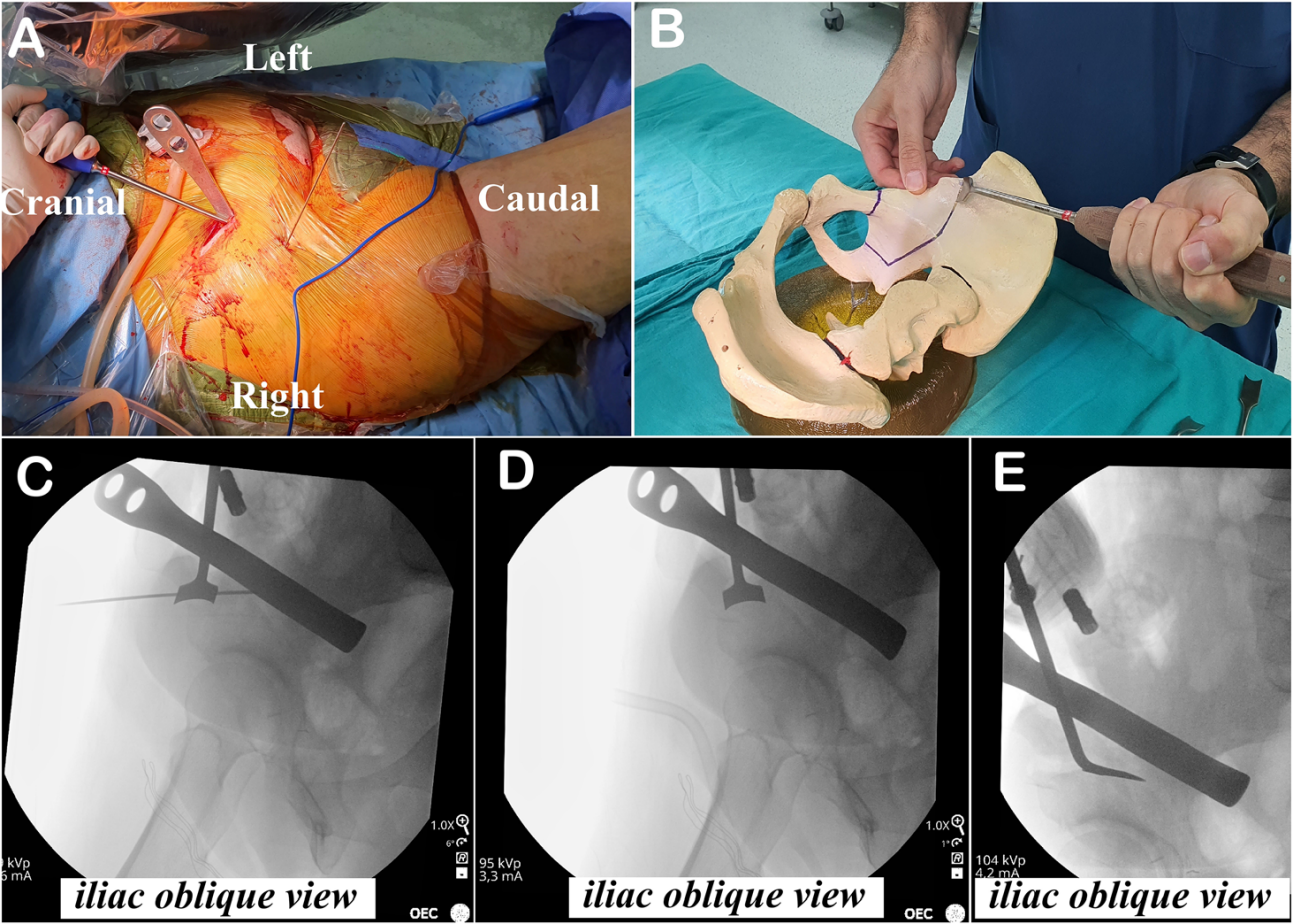
Supra‐acetabular iliac osteotomy with the patient positioned supine and the right hip as the operative side. (A) Soft‐tissue retraction with Hohmann retractors and positioning of the osteotome. (B) Presentation of the supra‐acetabular iliac osteotomy on a synthetic pelvic model. (C‐E) Sequential fluoroscopic views showing stepwise progression of the supra‐acetabular iliac osteotomy.

**FIGURE 7:**
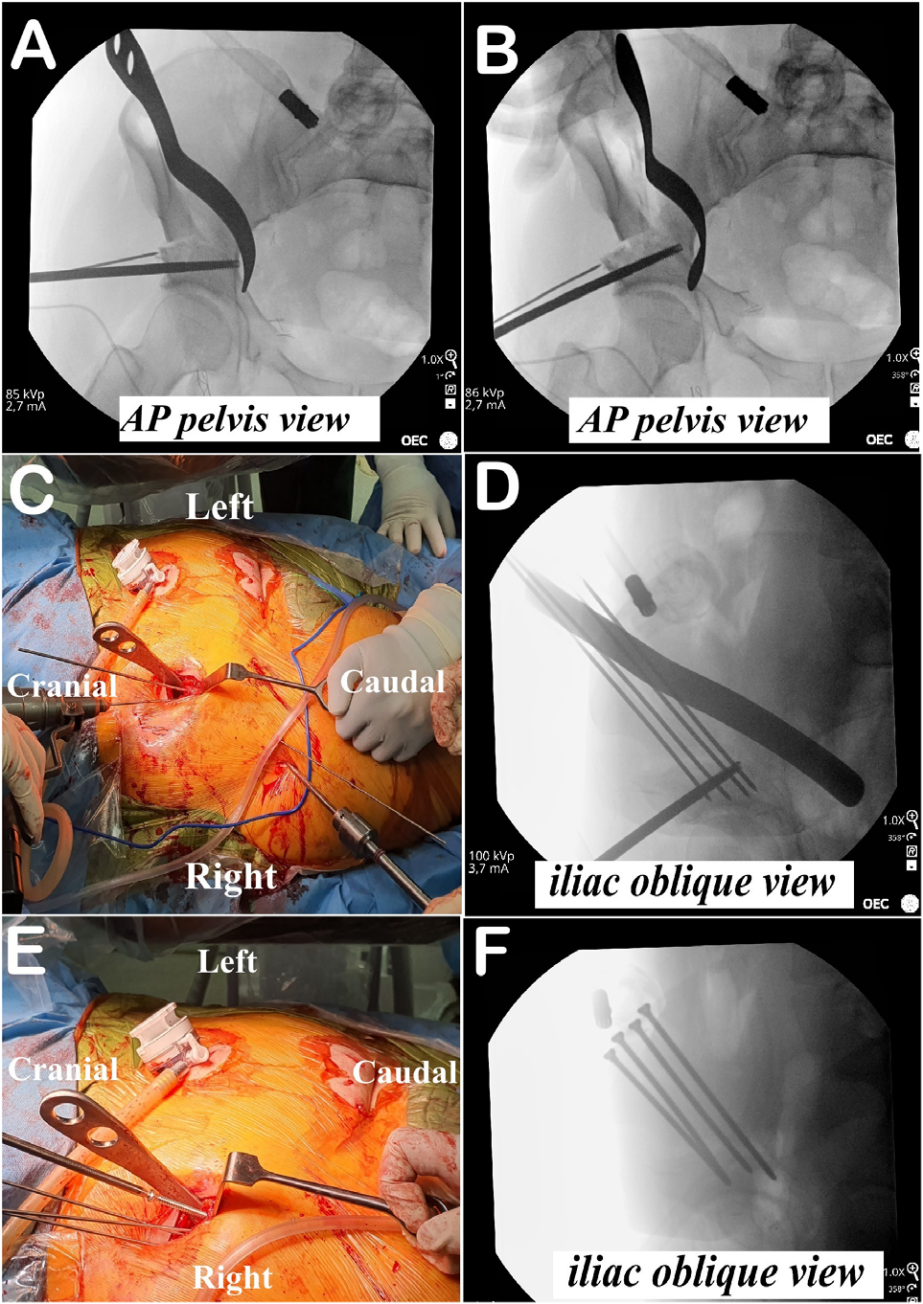
Acetabular reorientation and fixation with the right hip as the operative side. (A,B) Percutaneous placement of a Schanz screw and reorientation of the acetabular fragment using the screw as a joystick. (C,D) Fluoroscopic confirmation of satisfactory reorientation and provisional fixation with K‐wires. (E,F) Definitive fixation with appropriately sized screws.

**FIGURE 8:**
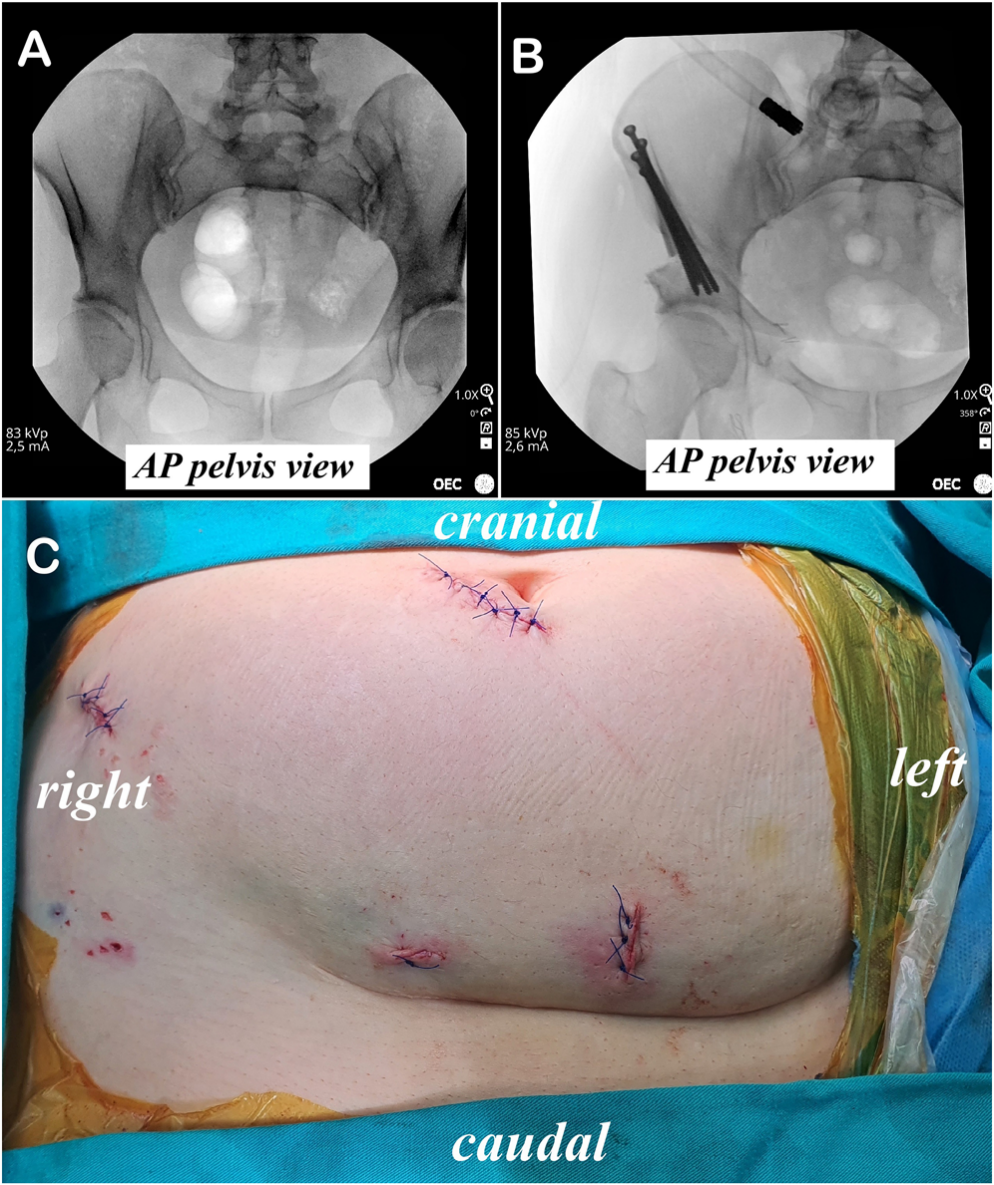
Preoperative and final assessment. (A) Preoperative AP pelvic fluoroscopic view. (B) Final AP pelvic fluoroscopic view after reorientation and fixation. (C) Postoperative appearance of the sutured incisions with the patient positioned supine and the right hip as the operative side. (AP, anteroposterior.)

### Preparation of the Preperitoneal Workspace

With the patient supine under general anesthesia, a 2‐ to 3‐cm crescent‐shaped incision is made 1 to 2 cm below the umbilicus for the infraumbilical visualization portal (Figure 1). The anterior rectus sheath is opened for 1 cm just lateral to the linea alba, the rectus abdominis is retracted laterally, and the plane between the anterior and posterior laminae of the rectus sheath is bluntly developed with the index finger.

A balloon dissector is introduced through this plane and inflated with 25 to 30 pumps to bluntly develop the preperitoneal space. A 10‐mm trocar is then inserted, and the space is insufflated with carbon dioxide at 10 to 15 mm Hg. A 10‐mm, 30° endoscope is advanced through the trocar. The anterior pelvic ring and preperitoneal retzius space are systematically evaluated (Figure 2A and 2B).

After the initial endoscopic assessment, a 5‐mm working portal for standard endoscopic instruments is planned to provide safe access and appropriate instrument angulation (Figure 1). Under endoscopic visualization, a percutaneous localization needle is used to confirm the entry point into the preperitoneal workspace and trajectory to the target (Figure 2C). A 5‐mm trocar is then inserted while avoiding injury to the inferior epigastric vessels coursing cranially along the anterior abdominal wall and to intraperitoneal structures (Figure 2D).

### Step 1: Ischial Osteotomy

The obturator neurovascular bundle is the principal structure at risk during this step and is exposed by blunt dissection from the superior pubic ramus toward the quadrilateral surface (Table 1). Any corona mortis encountered is clipped and divided with endoscopic scissors (Figure 2E and 2F).

**TABLE 1:** Pearls and Pitfalls of the O‐TEP PAO Technique

**Surgical Stage**	**Pearls**	**Pitfalls**
Preperitoneal workspace and portal placement	Use a percutaneous localization needle under endoscopic visualization to confirm the portal's entry point and trajectory before trocar insertion.	Incorrect portal placement or trajectory may injure the inferior epigastric vessels or intraperitoneal structures.
Ischial osteotomy	Identify the corona mortis and obturator neurovascular bundle before osteotomy. Position the osteotome inferior to the bundle and confirm its trajectory fluoroscopically. Perform the osteotomy transmuscularly through the obturator internus fibers without prior subperiosteal stripping.	Inadequate visualization of the obturator neurovascular bundle or incorrect osteotome trajectory may cause neurovascular injury or compromise posterior column integrity.
Pubic osteotomy	Retract the external iliac vessels laterally with a blunt retractor, position the osteotome endoscopically, and direct it medially away from the obturator neurovascular bundle.	Inadequate vascular retraction or excessive medial or lateral deviation of the osteotome may result in neurovascular injury.
Posterior column and supra‐acetabular iliac cuts	Confirm osteotome position and trajectory fluoroscopically, and advance each cut stepwise to connect with the preceding osteotomy.	Misdirected or incomplete cuts may prevent fragment mobilization or disrupt posterior column integrity.
Training and equipment	The procedure should be performed by surgeons experienced in both PAO and O‐TEP, with prior practical training in LAP‐TEP and access to appropriate endoscopic equipment.	Insufficient experience with extraperitoneal anatomy or endoscopic pelvic surgery may increase technical difficulty and complication risk.

LAP‐TEP, laparoscopic totally extraperitoneal; O‐TEP, orthopaedic total extraperitoneal; PAO, periacetabular osteotomy.

After exposure of the bundle, a 30° angled osteotome with a 20‐cm working length and 10‐mm cutting edge is introduced (Figure 3A). A 1‐cm osteotomy portal is created 3 cm cranial to the pubic symphysis and approximately 4 cm lateral to the midline contralateral to the operative side (Figure 1), and a 10‐mm trocar is gently inserted. Under endoscopic visualization, the osteotome is positioned inferior to the obturator neurovascular bundle (Figure 3B‐D) and adjusted under a 65° false‐profile fluoroscopic view to identify the anterior starting point of the cut (Figure 3E).

The osteotomy is performed transmuscularly through the obturator internus fibers without prior subperiosteal stripping to limit dissection and bleeding. The cut is completed from anterior to posterior in 2 or 3 sequential steps while preserving posterior column integrity (Figure 3E‐G).

### Step 2: Pubic Osteotomy

After completion of the ischial osteotomy, the second step is the pubic osteotomy (Figure 4A). The principal structures requiring protection during this step are the external iliac vessels and the obturator neurovascular bundle. The external iliac vessels are gently retracted laterally with a blunt retractor (Figure 4B and 4C); the osteotome is then positioned under direct endoscopic visualization, its position is confirmed on the obturator oblique view, and it is directed medially away from the bundle (Figure 4B‐D). The pubic cut is completed stepwise and probed with the osteotome to confirm completion (Figure 4E and 4F). After irrigation and hemostasis, the integrity of the obturator neurovascular bundle is reassessed endoscopically, and the endoscopic phase is concluded.

### Steps 3 and 4: Posterior Column and Supra‐acetabular Iliac Cuts

Both cuts are performed through the same lateral mini‐incision, which begins approximately 1 cm posterior to the anterior superior iliac spine and extends 3 to 4 cm posteriorly along the iliac crest (Figure 5A). Using an approach similar to iliac crest autograft harvest, the interval between the abdominal wall and hip abductors is developed, and subperiosteal dissection proceeds to the pelvic brim, where a Hohmann retractor is placed to retract and protect the iliopsoas complex and external iliac vessels (Figure 5A).

The posterior column cut is performed first, generally using a 10° angled osteotome with a 10‐mm cutting edge (Figure 5B). After fluoroscopic confirmation of its position and trajectory, the cut is advanced stepwise to connect with the ischial cut (Figure 5C‐E).

The final step is the supra‐acetabular iliac cut. The same lateral mini‐incision used for the posterior column cut is used for this step (Figure 6A). After appropriate retraction, a 40° angled osteotome with an 18‐mm cutting edge is positioned immediately superior to the anterior inferior iliac spine (Figure 6B). Under false‐profile fluoroscopy, its trajectory is aligned with the posterior column cut, and the osteotomy is completed in 2 or 3 sequential steps to connect with the posterior column cut (Figure 6C‐E).

### Acetabular Reorientation and Fixation

After completion of all cuts, a 6.5‐mm Schanz screw is inserted percutaneously from the lateral side and used as a joystick to mobilize and reorient the acetabular fragment (Figure 7A). Once satisfactory reorientation is confirmed fluoroscopically (Figure 7B), the fragment is provisionally fixed with K‐wires (Figure 7C and 7D) and definitively fixed with appropriately sized screws (Figure 7E and 7F). After definitive fixation and fluoroscopic confirmation (Figure 8A and 8B), the surgical field is irrigated, hemostasis is confirmed, and the wound is closed in layers (Figure 8C). Estimated blood loss in the index case was 400 mL, and no drain was placed.

### Ethics Approval

Because this manuscript is a Technical Note describing a surgical technique and does not involve a clinical research protocol or identifiable patient data, institutional ethics committee approval was not required. All radiographic, fluoroscopic, endoscopic, intraoperative, and postoperative images were obtained from the personal clinical archive of Vedat Öztürk, M.D., and were fully anonymized before submission. Written informed consent was obtained from the patient for the publication of the anonymized images and surgical video.

## DISCUSSION

The O‐TEP PAO technique preserves the standard Bernese osteotomy configuration while allowing the ischial and pubic cuts to be performed entirely through a portal‐based extraperitoneal endoscopic workspace. The posterior column and supra‐acetabular iliac cuts are completed through a lateral mini‐incision requiring limited dissection. Performing the ischial and pubic cuts endoscopically, together with positioning the mini‐incision posterior to the anterior superior iliac spine, avoids maneuvers that may be required in conventional anterior approaches, including rectus muscle elevation, detachment of the sartorius origin, anterior superior iliac spine osteotomy, and lateral femoral cutaneous nerve dissection (Table 2).

**TABLE 2:** Advantages, Risks, and Limitations of the O‐TEP PAO Technique

**Advantages**	**Risks**	**Limitations**
Direct endoscopic visualization facilitates identification and protection of the external iliac vessels, obturator neurovascular bundle, and corona mortis.	The risk of neurovascular injury cannot be completely eliminated despite direct endoscopic visualization.	The technique has a substantial learning curve and requires specialized endoscopic equipment and experience in both PAO and O‐TEP.
The ischial and pubic osteotomies are performed through portal‐based extraperitoneal endoscopic workspace, limiting soft‐tissue dissection and open surgical exposure.	Incorrect portal, trocar, or osteotome placement may injure vascular, neural, or intraperitoneal structures.	The sciatic nerve is not directly visualized during the procedure.
The posterior column and supra‐acetabular iliac cuts are completed through a limited lateral mini‐incision.	During the ischial osteotomy, the obturator neurovascular bundle is at risk and must be carefully identified and protected.	The technique is not fully endoscopic because the posterior column and supra‐acetabular iliac cuts are performed through a lateral mini‐incision.
The approach does not require rectus muscle elevation, sartorius‐origin detachment, ASIS osteotomy, or lateral femoral cutaneous nerve dissection.	During the pubic osteotomy, both the external iliac vessels and the obturator neurovascular bundle are at risk and should be clearly visualized, safely retracted, and protected.	The safety, reproducibility, learning curve, complication rates, and clinical outcomes have not yet been established in larger prospective comparative studies.
The standard 4‐cut Bernese PAO configuration is preserved.	Excessive advancement of the osteotome during the ischial cut may result in sciatic nerve injury.	
Limited soft‐tissue dissection and reduced open exposure may decrease postoperative pain and analgesic requirements, reduce surgical‐site infection risk, and facilitate earlier mobilization, rehabilitation, and hospital discharge.		

ASIS, anterior superior iliac spine; O‐TEP, orthopaedic total extraperitoneal; PAO, periacetabular osteotomy.

Inan et al. described an endoscopy‐assisted curvilinear PAO in a cadaveric model; however, the osteotomies were performed using instruments introduced through a miniopen incision.^3^ In the technique described by Matsuda et al., arthroscopy and endoscopy were used as adjuncts to miniopen PAO for protecting the sciatic nerve and visualizing selected osteotomy steps.^4^, ^10^, ^15^ Open anterior intrapelvic approaches provide direct visualization of the osteotomy surfaces and anatomical structures at risk but require more extensive surgical exposure.^11^

The principal potential advantage of O‐TEP PAO is that it enables direct endoscopic visualization and protection of anterior intrapelvic structures at risk without requiring extensive surgical exposure. However, whether these theoretical and technical advantages translate into lower complication rates or improved clinical outcomes remains unknown.

The technique has a substantial learning curve and requires specialized endoscopic equipment and experience in both PAO and O‐TEP. The risk of neurovascular injury cannot be completely eliminated despite direct visualization. In addition, the sciatic nerve is not directly visualized, and the posterior column and supra‐acetabular iliac cuts are performed through a lateral mini‐incision. Therefore, O‐TEP PAO is not a fully endoscopic technique; however, showing that the intrapelvic steps of PAO can be performed through a portal‐based workspace may provide a conceptual and technical foundation for the development of more comprehensive endoscopic techniques. Because this report is a technical description, the safety, reproducibility, learning curve, and clinical outcomes of the technique should be evaluated in larger prospective comparative studies.

## DECLARATION OF GENERATIVE AI AND AI‐ASSISTED TECHNOLOGIES IN THE WRITING PROCESS

During the preparation of this work, the authors used ChatGPT (OpenAI) solely for English‐language translation and language editing. After using this tool/service, the authors reviewed and edited the content as needed and take full responsibility for the content of the publication.

## DISCLOSURES

The authors (V.Ö., M.Ç., S.Ç., Ö.M.H., F.Ç.A., C.K., M.G.B.) declare that they have no known competing financial interests or personal relationships that could have appeared to influence the work reported in this article.
